# Draft genomic sequence of a chromate- and sulfate-reducing *Alishewanella* strain with the ability to bioremediate Cr and Cd contamination

**DOI:** 10.1186/s40793-016-0169-3

**Published:** 2016-08-05

**Authors:** Xian Xia, Jiahong Li, Shuijiao Liao, Gaoting Zhou, Hui Wang, Liqiong Li, Biao Xu, Gejiao Wang

**Affiliations:** 1 State Key Laboratory of Agricultural Microbiology, Huazhong Agricultural University, Wuhan, 430070 People’s Republic of China; 2 College of Basic Sciences, Huazhong Agricultural University, Wuhan, 430070 People’s Republic of China

**Keywords:** *Alishewanella*, Chromate-reducing bacterium, Sulfate-reducing bacterium, Cadmium, Chromium

## Abstract

*Alishewanella* sp. WH16-1 (= CCTCC M201507) is a facultative anaerobic, motile, Gram-negative, rod-shaped bacterium isolated from soil of a copper and iron mine. This strain efficiently reduces chromate (Cr^6+^) to the much less toxic Cr^3+^. In addition, it reduces sulfate (SO_4_^2−^) to S^2−^. The S^2−^ could react with Cd^2+^ to generate precipitated CdS. Thus, strain WH16-1 shows a great potential to bioremediate Cr and Cd contaimination. Here we describe the features of this organism, together with the draft genome and comparative genomic results among strain WH16-1 and other *Alishewanella* strains. The genome comprises 3,488,867 bp, 50.4 % G + C content, 3,132 protein-coding genes and 80 RNA genes. Both putative chromate- and sulfate-reducing genes are identified.

**Electronic supplementary material:**

The online version of this article (doi:10.1186/s40793-016-0169-3) contains supplementary material, which is available to authorized users.

## Introduction

The genus *Alishewanella* was established by Vogel et al., in 2000 with *Alishewanella fetalis* as the type species. It belongs to the family *Alteromonadaceae* of the class *Gammaproteobacteria* [1]*.* So far, *Alishewanella* contains six species: *A. fetalis*, *Alishewanella aestuarii*, *Alishewanella jeotgali*, *Alishewanella agri* and *Alishewanella tabrizica* and *Alishewanella solinquinati* [1–6]. The common characteristics of the genus *Alishewanella* are Gram-negative, rod-shaped and positive for oxidase and catalase [1–6]. Some *Alishewanella* strains were able to degrade pectin which is applicable in bioremediation of food industrial wastes [7–11]. Three *Alishewanella* strains (*A. aestuarii* B11^T^*,**A. jeotgali*KCTC 22429^T^ and *A. agri* BL06^T^) have been sequenced and the pectin degradation pathway was found in their genomes [8–11]. Some strains of *Alishewanella* were reported to tolerate arsenic [12, 13], but the ability of *Alishewanella* strains to resist or transform other heavy metal(loids) have not been reported.

*Alishewanella* sp. WH16-1 was isolated from mining soil in 2009. This strain could resist to multiple heavy metals. During cultivation, it could efficiently reduce the toxic chromate (Cr^6+^) to the much less toxic and less bioavaliable Cr^3+^. It could also reduce sulfate (SO_4_^2−^) to S^2−^. When Cd^2+^ was present, the S^2−^ reacted with Cd^2+^ and precipitated as CdS. These characteristics made strain WH16-1 a great potential for bioremediate Cr and Cd contamination. In pot experiments of rice, tobacco and Chinese cabbage, with the addition of the bacterial culture, the amount of Cr and Cd in the plants decreased significantly [14]. Sequencing the genome of WH16-1 and comparing its attributes with the other *Alishewanella* genomes would provide a means of establishing the molecular determinants required for chromate/sulfate reduction, heavy metal resistance and pectin degradation, and for better application of these strains. Here we report the high quality draft genomic information of strain WH16-1 and compare it to the three sequenced *Alishewanella* genomes.

## Organism information

### Classification and features

**Fig. 1:**
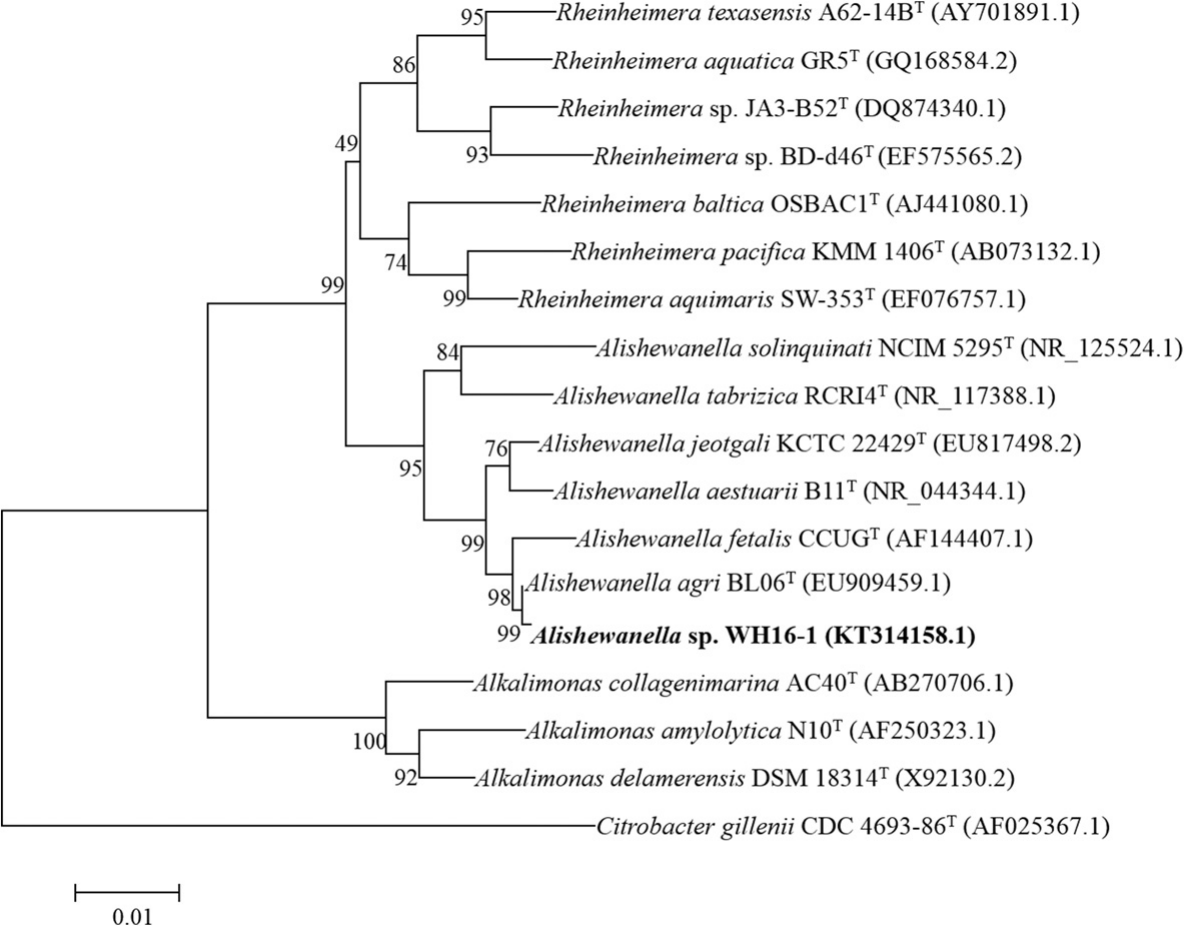
Phylogenetic tree highlighting the phylogenetic position of *Alishewanella* sp. WH16-1. The phylogenetic tree was constructed based on the 16S rRNA gene sequences. The analysis was inferred by MEGA 6.0 [45] with NJ algorithm and 1,000 bootstrap repetitions were computed to estimate the reliability of the tree

Phylogenetic analysis was performed by the neighbor-joining method based on 16S rRNA gene sequences. Strain WH16-1 is closely related to *A. agri* BL06^T^ (99.7 %) and *A. fetalis*CCUG 30811^T^ (99.1 %) (Fig. 1). A similar result was obtained based on gyrase B gene (*gyrB)* sequences (Additional file 1: Figure S1). The *gyrB* sequences has been successfully used to establish phylogenetic relatedness in *Alishewanella* [1], *Pseudomonas* [15], *Acinetobacter* [16], *Vibrio* [17]*,**Bacillus* [18] and *Shewanella* [19].

**Fig. 2:**
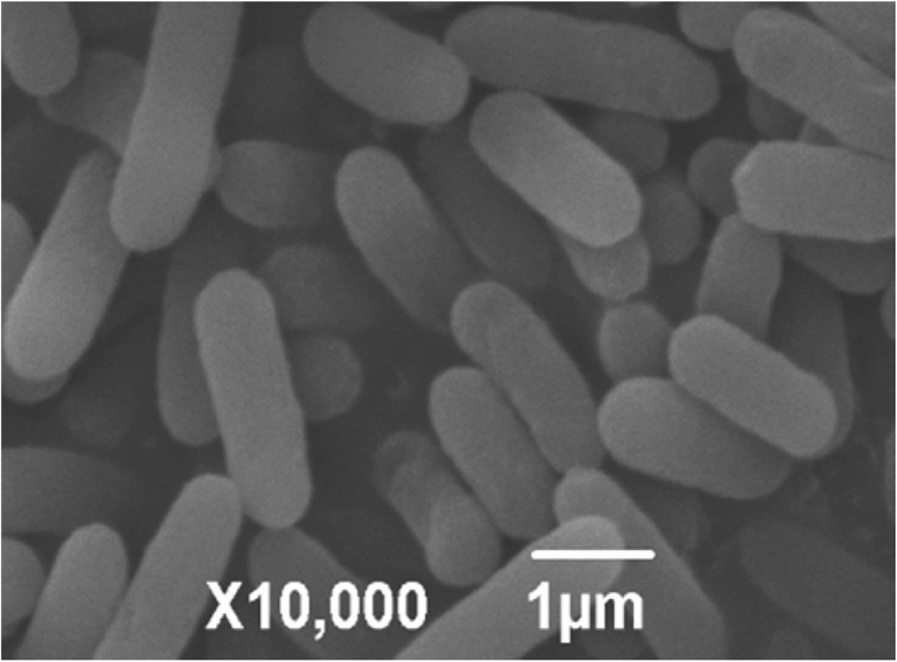
Scan electron microscope (SEM) image of *Alishewanella* sp. WH16-1 cells. The bar scale represents 1 μm

Strain WH16-1 is Gram-negative, facultatively anaerobic, motile and rod-shaped (0.3–0.5 × 1.2–2.0) (Fig. 2). Colonies are white, circular and raised on LB agar plate. Growth occurs at 4–40 °C, in 0–8 % (w/v) NaCl and at pH 4–11. Optimal growth occurs at 37 °C, 1 % (w/v) NaCl and at pH 6.0–8.0 (Table 1). It can grow in LB, trypticase soy broth and R2A medium. API 20NE test (bioMérieux) in combination of traditional classification methods were used to analyze the physiological and biochemical characteristics. Strain WH16-1 is positive for oxidase and catalase activities and is able to reduce nitrate to nitrite. It is positive for aesculinase, gelatinase, arginine dihydrolase and urease but is negative for indole and β-galactosidase. It can use D-sucrose and maltose as the sole carbon sources. It cannot assimilate D-glucose, L-arabinose, D-mannose, D-mannitol, N-acetylglucosamine, gluconate, capric acid, adipic acid, malic acid, trisodium citrate or phenylacetic acid. Most of these biochemical characteristics are similar to the other *Alishewanella* strains [1–6]. However, unlike some *Alishewanella* strains [8–11], strain WH16-1 cannot degrade pectin.

**Table 1:** Classification and general features of *Alishewanella* sp. WH16-1 [47]

MIGS ID	Property	Term	Evidence code^a^
	Classification	Domain *Bacteria*	TAS [48]
		Phylum *Proteobacteria*	TAS [49, 50]
		Class *Gammaproteobacteria*	TAS [51–53]
		Order *Alteromonadales*	TAS [52–54]
		Family *Alteromonadaceae*	TAS [55]
		Genus *Alishewanella* Species *Alishewanella* sp.	TAS [1]
		Strain WH16-1	
	Gram stain	negative	IDA
	Cell shape	rod	IDA
	Motility	motile	IDA
	Sporulation	non-sporulating	NAS
	Temperature range	4–40 °C	IDA
	Optimum temperature	37 °C	IDA
	pH range; Optimum	4–11; 6–8	IDA
	Carbon source	maltose, D-sucrose	IDA
MIGS-6	Habitat	soil	IDA
MIGS-6.3	Salinity	0–8 % NaCl (w/v), optimal at 1 %	IDA
MIGS-22	Oxygen requirement	facultative anaerobic	IDA
MIGS-15	Biotic relationship	free-living	IDA
MIGS-14	Pathogenicity	non-pathogen	NAS
MIGS-4	Geographic location	Huangshi city, Hubei province, China	IDA
MIGS-5	Sample collection	2009	IDA
MIGS-4.1	Latitude	N29°40′–30°15′	IDA
MIGS-4.2	Longitude	E114°31′–115°20′	IDA
MIGS-4.4	Altitude	not reported	

These evidence codes are from the Gene Ontology project [56]

*IDA* Inferred from Direct Assay, *TAS* Traceable Author Statement (i.e., a direct report exists in the literature), *NAS* Non-traceable Author Statement (i.e., not directly observed for the living, isolated sample, but based on a generally accepted property for the species, or anecdotal evidence)

^a^ Evidence codes

**Fig. 3:**
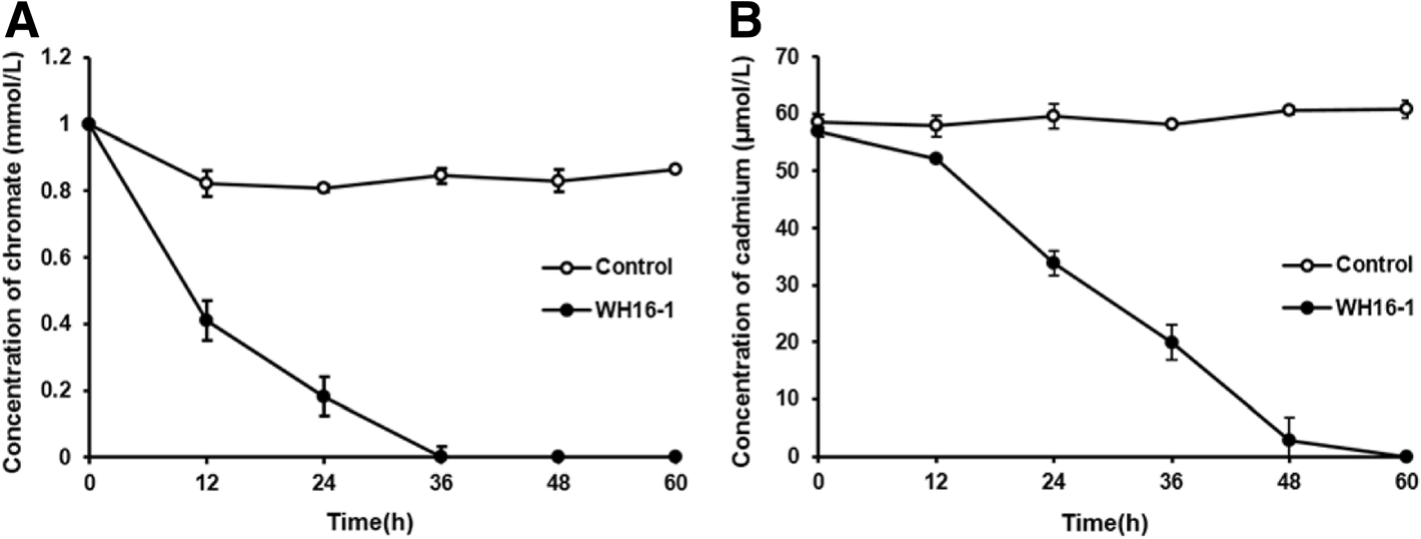
Cr^6+^ and Cd^2+^ removed by *Alishewanella* sp. WH16-1. Control stands for null LB medium. Strain WH16-1 was incubated until OD_600_ reach 1.0, and then amended with K_2_CrO_4_ (1 mmol/L) and CdCl_2_ (0.06 mmol/L), respectively. The cultures were removed at 12 h intervals. After centrifuging at 12,000 rpm for 2 min, the supernatant was used to determine the residual concentration of Cr^6+^ and Cd^2+^. The concentration of Cr^6+^ and Cd^2+^ were measured by the UV spectrophotometer (DU800, Beckman, CA, USA) with the colorimetric diphenylcarbazide (DPC) method [46] and the atomic absorption spectrometry AAS, respectively

Interestingly, the strain could reduce 1 mmol/L Cr^6+^ (added as K_2_CrO_4_) in 36 h and remove 60 μmol/L Cd^2+^ (added as CdCl_2_) in 60 h (by the production of precipitated CdS [20] in LB liquid medium) (Fig. 3). In addition, this strain is tolerant to multi-metal(loids). The minimal inhibition concentration tests for different heavy metals were carried out on LB agar plates and incubated at 37 °C for 2 days. The MICs for K_2_CrO_4_, CdCl_2_, PbCl_2_, CuCl_2_ and Na_3_AsO_3_ are 45, 0.08, 10, 1 and 1 mmol/L, respectively.

## Genome sequencing information

### Genome project history

Strain WH16-1 was selected for genome sequencing based on its ability to reduce Cr^6+^ and SO_4_^2−^ and preliminary application for soil Cr and Cd bioremediation. Since 2009, this strain has been used in both basic and bioremediation studies and the results are very promising. It was sequenced by Majorbio Bio-pharm Technology Co., Ltd, Shanghai, China. The genome sequencing and assembly information of the project is given in Table 2. The final genome consists of 133 scaffolds with approximately 345.3 × coverage. The draft genome sequence was annotated by NCBI PGAP. The genome sequence is available in DDBJ/EMBL/GenBank under accession number LCWL00000000.

**Table 2:** Project information

MIGS ID	Property	Term
MIGS-31	Finishing quality	High-quality draft
MIGS-28	Libraries used	Illumina Paired-End library (300 bp insert size)
MIGS-29	Sequencing platforms	Illumina Hiseq 2000
MIGS-31.2	Fold coverage	345.3 ×
MIGS-30	Assemblers	SOAPdenovo v1.05
MIGS-32	Gene calling method	GeneMarkS^+^
	Locus TAG	AAY72
	Genbank ID	LCWL00000000
	Genbank Date of Release	2015.11.12
	Bioproject	PRJNA283029
MIGS-13	Source material identifier	Strain CCTCC M201507
	Project relevance	Bioremediation

## Growth conditions and genomic DNA preparation

A single colony of strain WH16-1 was incubated into 50 ml LB medium and grown aerobically at 37 °C for 36 h with 150 rpm shaking. The cells were collected by centrifugation. The DNA was extracted, concentrated and purified using the QiAamp kit (Qiagen, Germany). A NanoDrop Spectrophotometer 2000 was used to determine the quality and quantity of the DNA. Six micrograms of DNA was sent to Majorbio Bio-pharm Technology Co., Ltd (Shanghai, China) for sequencing.

### Genome sequencing and assembly

The genome sequencing of strain WH16-1 was performed on an Illumina Hiseq2000 [21] and assembled by Majorbio Bio-pharm Technology Co., Ltd, Shanghai, China. An Illumina standard shotgun library was constructed and sequenced, which generated 12,683,662 reads totaling 1,281,049,862 bp. All original sequence data can be found at the NCBI Sequence Read Archive [22]. The following steps were performed for removing low quality reads: (1) removed the adapter o reads; (2) cut the 5′ end bases which were not A, T, G, C; (3) filtered the reads which have a quality score lower than 20; (4) filtered the reads which contained N more than 10 %; and (5) removed the reads which have the length less than 25 bp after processed by the previous four steps. The reads were assembled into 156 contigs using SOAPdenovo v1.05 [23]. A total of 149 contigs were obtained after removing the contigs < 200 bp. The total size of the genome is 3,488,867 bp and the final assembly is based on 1,205 Mbp of Illumina data which provides a coverage of 345.3 × .

### Genome annotation

The draft genome of WH16-1 was annotated through the NCBI PGAP, which combines the gene caller GeneMarkS^+^ [24] with the similarity-based gene detection approach. Protein function classification was performed by WebMGA [25] with E-value cutoff of 1-e10. The transmembrane helices were predicted by TMHMM v. 2.0 [26]. Signal peptides in the genome were predicted by SignalP 4.1 [27]. The translations of the predicted CDSs were also used to search against the Pfam protein family database with E-value cutoff of 1-e5 [28] and the KEGG database [29]. Internal gene clustering was performed by OrthoMCL using Match cutoff of 50 % and E-value Exponent cutoff of 1-e5 [30, 31].

## Genome properties

The whole genome of strain WH16-1 is 3,488,867 bp in length, with an average G + C content of 50.4 %, and is distributed in 149 contigs (>200 bp). The genome properties and statistics are summarized in Table 3. There are 80 predicted RNA including 73 tRNA, 5 rRNAs and 2 ncRNA. In addition, a total of 3,132 protein-coding genes are identified. The distribution of genes into COGs functional categories is presented in Table 4.

**Table 3:** Nucleotide content and gene count levels of the genome

Attribute	Genome (total)	
	Value	% of total^a^
Genome size (bp)	3,488,867	100.00
DNA coding (bp)	3,117,033	89.34
DNA G + C (bp)	1,759,785	50.44
DNA scaffolds	133	100.00
Contigs	149	100.00
Total genes^b^	3,282	
RNA genes	80	
Pseudo genes	73	
Protein-coding genes	3,132	100.00
Genes in internal clusters	1,190	37.99
Genes with function prediction	2,388	76.25
Genes assigned to COGs	2,249	71.81
Genes with Pfam domains	2,710	86.53
Genes with signal peptides	367	11.72
Genes with transmembrane helices	1,101	35.15
CRISPR repeats	1	

^a^The total is based on either the size of the genome in base pairs or the total number of protein coding genes in the annotated genome

^b^Also includes 73 pseudogenes, 73 tRNA genes, 5 rRNAs and 2 ncRNA

**Table 4:** Number of genes associated with the 25 general COG functional categories

Code	Value	% of total^a^	Description
J	175	5.59	Translation
A	1	0.03	RNA processing and modification
K	153	4.89	Transcription
L	141	4.50	Replication, recombination and repair
B	2	0.06	Chromatin structure and dynamics
D	34	1.09	Cell cycle control, mitosis and meiosis
Y	0	0.00	Nuclear structure
V	56	1.79	Defense mechanisms
T	216	6.90	Signal transduction mechanisms
M	156	4.98	Cell wall/membrane biogenesis
N	87	2.78	Cell motility
Z	0	0.00	Cytoskeleton
W	0	0.00	Extracellular structures
U	77	2.46	Intracellular trafficking and secretion
O	116	3.70	Posttranslational modification, protein turnover, chaperones
C	157	5.01	Energy production and conversion
G	90	2.87	Carbohydrate transport and metabolism
E	207	6.61	Amino acid transport and metabolism
F	62	1.98	Nucleotide transport and metabolism
H	132	4.21	Coenzyme transport and metabolism
I	85	2.71	Lipid transport and metabolism
P	148	4.73	Inorganic ion transport and metabolism
Q	41	1.31	Secondary metabolites biosynthesis, transport and catabolism
R	244	7.79	General function prediction only
S	202	6.45	Function unknown
-	885	28.26	Not in COGs

^a^The total is based on the total number of protein coding genes in the annotated genome

## Insights from the genome sequence

Strain WH16-1 has the genes for a complete SO_4_^2−^ reduction pathway according to the KEGG analysis, including CysPUWA, CysN, CysD, CysC, CysH and CysIJ (Additional file 1: Figure S2; Additional file 2: Table S1). This pathway contained several steps: 1) the SO_4_^2−^ is uptaken by the putative CysPUWA into the cell [32]; 2) the intracellular SO_4_^2−^ is acetylated to adenylylsulphate (APS) by sulfate adenylyltransferases CysN and CysD [33]; 3) the APS is phosphorylated to phosphoadenylylsulphate (PAPS) by APS kinase CysC and, 4) the PAPS is reduced to sulfite (SO_3_^2−^) by PAPS reductase CysH [33] and, 5) the SO_3_^2−^ is finally reduced to sulfide (S^2−^) by sulfite reductase CysIJ [33]. Strain WH16-1 was able to remove Cd^2+^ most probably due to the reaction between S^2−^ and Cd^2+^ to form the precipitated CdS [20]. For Cr^6+^ reduction, a putative chromate reductase YieF was found (Additional file 2: Table S1). YieF was reported to responsible for the reduction of Cr^6+^ in cytoplasm [34]. An individual chromate transport gene *chrA* and a chromate resistance cluster including *chrBAC*, *hp1*, *chrF*, *lppy*/*lpqo*, *hp2* and *ABC transport permease* gene are found in the genome (Additional file 2: Table S2) [35, 36]. Currently, we have disrupted the *chrA* (AAY72_02075) and the *ABC transport permease* genes, respectively. The chromate resistance levels were both decreased significantly in the *chrA* and *ABC transport permease* gene mutant strains (data not shown).

In addition, various heavy metal transformation and resistance determinants are identified in the genome of strain WH16-1 Several transporters (MntH, CzcA and ZntA) that might be involved in the efflux of Cd^2+^, Pb^2+^ and Zn^2+^ are found [37–39]. Cu^2+^, As^3+^ and Hg^2+^ resistance determinants are also present, such as Cu transporter ATPase [40], Cu^2+^ resistance system CopABCD [41], Ars [42] and Pst [43] systems for arsenic resistance and MerTPADE system for mercury resistance [44] (Additional file 2: Table S2).

**Fig. 4:**
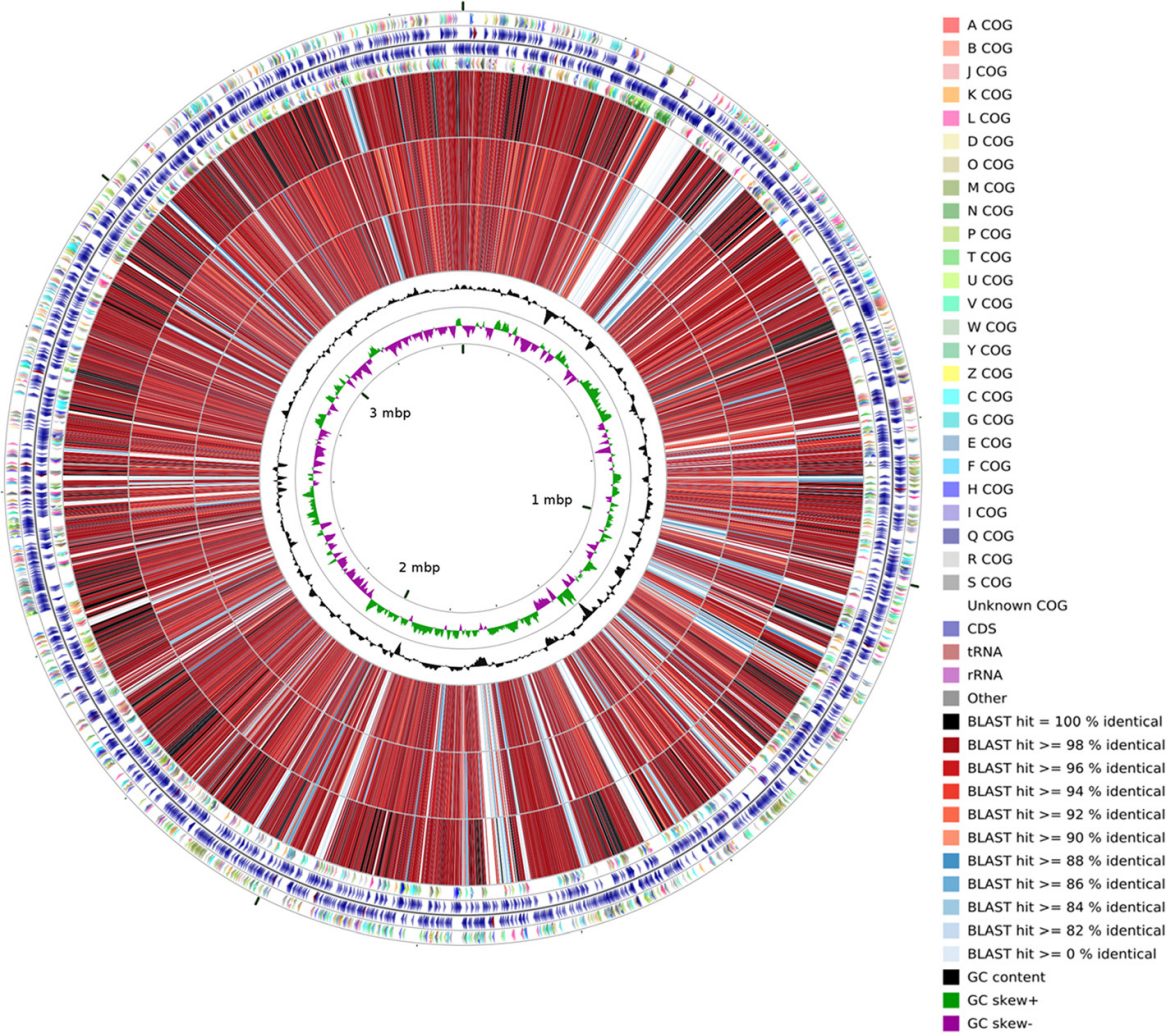
A graphical circular map of the comparison between reference strain *Alishewanella* sp. WH16-1 and three sequenced strains of the *Alishewanella* species. From outside to center, rings 1, 4 show protein-coding genes colored by COG categories on forward/reverse strand; rings 2, 3 denote genes on forward/reverse strand; rings 5, 6, 7 show the CDS vs CDS BLAST results of strain WH16-1 with those of *A. agri* BL06^T^, *A. jeotgali* KCTC 22429^T^ and *A. aestuarii* B11^T^, respectively; ring 8 shows G + C % content plot and the innermost ring shows GC skew

**Fig. 5:**
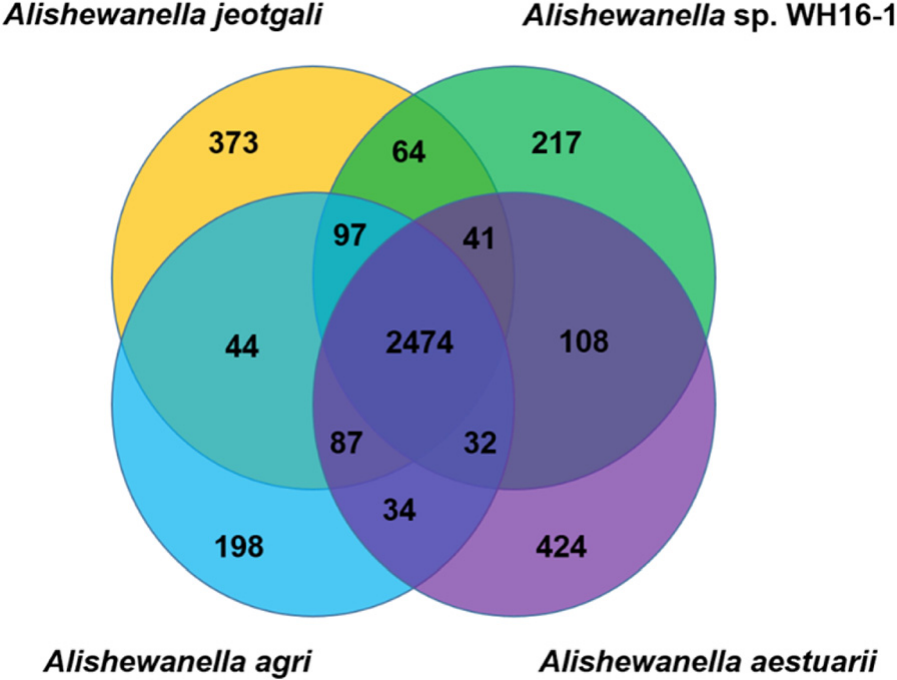
The Venn diagram depicting the core and unique genes between *Alishewanella* sp. WH16-1 and other three *Alishewanella* species (*A. agri* BL06^T^, *A. jeotgali* KCTC 22429^T^ and *A. aestuarii* B11^T^)

Strain WH16-1 has a genome size (3.49 Mbp), similar to *A. jeotgali*KCTC 22429^T^ (3.84 Mbp), *A. aestuarii* B11^T^ (3.59 Mbp) and *A. agri* BL06^T^ (3.49 Mbp) [8–10] (Fig. 4). The G + C content of strain WH16-1 (50.4 %) is also consistent with the other *Alishewanella* strains (*A. jeotgali*KCTC 22429^T^, 50.7 %, *A. aestuarii* B11^T^, 51 % and *A. agri* BL06^T^, 50.6 %). Strain WH16-1 shares 2,474 proteins with the other three *Alishewanella* genomes and has 217 strain-specific proteins (Fig. 5). The 2,474 core genes include *yieF*, *chrA*, the ten genes in the whole sulfate reduction pathway and most of the heavy metal resistance genes (Additional file 2: Table S1-S2). Strain WH16-1 possesses the higher number of chromatin resistance genes compared to the other three strains.

In addition, *A. agri* BL06^T^, *A. jeotgali*KCTC 22429^T^ and *A. aestuarii* B11^T^ were all reported to have the ability of degrading pectin and possess pectin degradation genes [8–11]. However, unlike strains BL06^T^, KCTC 22429^T^ and B11^T^, strain WH16-1 was unable to degrade pectin and the pectin degradation genes are not found in its genome. Since strain WH16-1 was isolated from a heavy metal rich environment, it may be more relevant for bioremediation of heavy metal contamination. The pectin degradation genes may be lost during the evolution.

## Conclusions

The genomic results of *Alishewanella* sp. WH16-1 reveal correlation between the gene types and some phenotypes. The strain harbors various genes responsible for sulfate transport and reduction, chromate reduction and resistance of multi-heavy metals. These observations provide insights into understand the molecular mechanisms of heavy metals. In addition, all of the analyzed *Alishewanella* genomes have putative sulfate and chromate reduction genes, which indicates that sulfate and chromate reduction may be the important characters of the *Alishewanella* strains. Thus, these strains have a great potential for application in bioremediation of heavy metal or other industrial wastes.

## Additional files

Additional file 1:
**Figure S1.** Phylogenetic relationships of *Alishewanella* sp. WH16-1 based on *gyrB* sequences. The analysis was performed by MEGA 6.0 [45] with NJ algorithm and 1,000 bootstrap repetitions were computed to estimate the reliability of the tree. The *gyrB* gene of strain WH16-1 is the gene sequence coding for AAY72_13600. **Figure S2.** The putative sulfate transport and reduction pathway in *Alishewanella* sp. WH16-1. APS stands for adenylylsulphate, PAPS stands for phosphoadenylylsulphate. The locus tag numbers of the predicted proteins (CysP, CysU, CysW, CysA, CysN, CysD, CysC, CysH CysJ and CysI) are AAY72_14890, AAY72_14885, AAY72_14880, AAY72_14875, AAY72_03865, AAY72_03870, AAY72_07290, AAY72_07265, AAY72_07255 and AAY72_07260, respectively. (DOCX 355 kb) Additional file 2:
**Table S1.** Putative proteins involved in sulfate and chromate reduction. **Table S2.** Putative proteins involved in heavy metal resistance. (XLSX 14 kb)

## Abbreviation

PGAP: Prokaryotic Genome Annotation Pipeline

## References

[1] Vogel BF, Venkateswaran K, Christensen H, Falsen E, Christiansen G, Gram L (2000). Polyphasic taxonomic approach in the description of *Alishewanella fetalis* gen. nov., sp. nov., isolated from a human foetus. Int J Syst Evol Microbiol.

[2] Roh SW, Nam YD, Chang HW, Kim KH, Kim SM, Oh HM, Bae JW (2009). *Alishewanella aestuarii* sp. nov., isolated from tidal flat sediment, and emended description of the genus *Alishewanella*. Int J Syst Evol Microbiol.

[3] Kim MS, Roh SW, Nam YD, Chang HW, Kim KH, Jung MJ (2009). *Alishewanella jeotgali* sp. nov., isolated from traditional fermented food, and emended description of the genus *Alishewanella*. Int J Syst Evol Microbiol.

[4] Kim MS, Jo SK, Roh SW, Bae JW (2010). *Alishewanella agri* sp. nov., isolated from landfill soil. Int J Syst Evol Microbiol.

[5] Tarhriz V, Nematzadeh G, Vahed SZ, Hejazi MA, Hejazi MS (2012). *Alishewanella tabrizica* sp. nov., isolated from Qurugöl Lake. Int J Syst Evol Microbiol.

[6] Kolekar YM, Pawar SP, Adav SS, Zheng LQ, Li WJ (2013). *Alishewanella solinquinati* sp. nov., isolated from soil contaminated with textile dyes. Curr Microbiol.

[7] Miran W, Nawaz M, Jang J, Lee DS (2016). Conversion of orange peel waste biomass to bioelectricity using a mediator-less microbial fuel cell. Sci Total Environ.

[8] Jung J, Choi S, Chun J, Park W (2012). Genome sequence of pectin-degrading *Alishewanella aestuarii* strain B11^T^, isolated from tidal flat sediment. J Bacteriol.

[9] Kim J, Jung J, Sung JS, Chun J, Park W (2012). Genome sequence of pectin-degrading *Alishewanella agri*, isolated from landfill soil. J Bacteriol.

[10] Jung J, Chun J, Park W (2012). Genome sequence of extracellular-protease-producing *Alishewanella jeotgali* isolated from traditional Korean fermented seafood. J Bacteriol.

[11] Jung J, Park W (2013). Comparative genomic and transcriptomic analyses reveal habitat differentiation and different transcriptional responses during pectin metabolism in *Alishewanella* species. Appl Environ Microbiol.

[12] Shah R, Jha S (2013). *Alishewanella* sp. strain GIDC-5, Arsenite hyper-tolerant bacteria isolated from industrial effluent of South Gujarat, India. Chem Ecol.

[13] Li P, Wang Y, Dai X, Zhang R, Jiang Z, Jiang D (2015). Microbial community in high arsenic shallow groundwater aquifers in Hetao Basin of Inner Mongolia, China. PLoS One.

[14] Liao S, Wang G, Zhou G, Xia X, Wang H. An *Alishewanella* strain with the ability to bioremediate heavy metal contamination. China Patent. 2015; CN 104,928,213 A.

[15] Yamamoto S, Harayama S (1995). PCR Amplification and direct sequencing of *gyrB* genes with universal primers and their application to the detection and taxonomic analysis of *Pseudomonas putida s*trains. Appl Environ Microbiol.

[16] Yamamoto S, Harayama S (1996). Phylogenetic analysis of *Acinetobacter* strains based on the nucleotide sequences of *gyrB* genes and on the amino acid sequences of their products. Int J Syst Bacteriol.

[17] Venkateswaran K, Dohmoto N, Harayama S (1998). Cloning and nucleotide sequence of the *gyrB* gene of *Vibrio parahaemolyticus* and its application in detection of this pathogen in shrimp. Appl Environ Microbiol.

[18] Yamada S, Ohashi E, Agata N, Venkateswaran K (1999). Cloning and nucleotide sequence analysis of *gyrB* of *Bacillus cereus*, *B. thuringiensis*, *B. mycoides*, and *B. anthracis* and their application to the detection of *B. cereus* in rice. Appl Environ Microbiol.

[19] Venkateswaran K, Moser DP, Dollhopf ME, Lies DP, Saffarini DA, MacGregor BJ (1999). Polyphasic taxonomy of the genus *Shewanella* and description of *Shewanella oneidensis* sp. nov. Int J Syst Bacteriol.

[20] Pagnanelli F, Cruz Viggi C, Toro L (2010). Isolation and quantification of cadmium removal mechanisms in batch reactors inoculated by sulphate reducing bacteria: biosorption versus bioprecipitation. Bioresour Technol.

[21] Bennett S (2004). Solexa Ltd. Pharmacogenomics.

[22] The NCBI Sequence Read Archive (SRA). [http://www.ncbi.nlm.nih.gov/Traces/sra/].

[23] Li R, Li Y, Kristiansen K, Wang J (2008). SOAP: short oligonucleotide alignment program. Bioinformatics.

[24] Besemer J, Lomsadze A, Borodovsky M (2001). GeneMarkS: a self-training method for prediction of gene starts in microbial genomes. Implications for finding sequence motifs in regulatory regions. Nucleic Acids Res.

[25] Wu S, Zhu Z, Fu L, Niu B, Li W (2011). WebMGA: a customizable web server for fast metagenomic sequence analysis. BMC Genomics.

[26] Krogh A, Larsson BÈ, Von Heijne G, Sonnhammer EL (2001). Predicting transmembrane protein topology with a hidden Markov model: application to complete genomes. J Mol Biol.

[27] Petersen TN, Brunak S, Heijne GV, Nielsen H (2011). SignalP 4.0: discriminating signal peptides from transmembrane regions. Nat Methods.

[28] Finn RD, Bateman A, Clements J, Coggill P, Eberhardt RY, Eddy SR (2014). Pfam: the protein families database. Nucleic Acids Res.

[29] Kanehisa M, Goto S, Kawashima S, Okuno Y, Hattori M (2004). The KEGG resource for deciphering the genome. Nucleic Acids Res.

[30] Li L, Stoeckert CJ, Roos DS (2003). OrthoMCL: identification of ortholog groups for eukaryotic genomes. Genome Res.

[31] Fischer S, Brunk B P, Chen F, Gao X, Harb OS, Iodice JB, et al. Using OrthoMCL to assign proteins to OrthoMCL-DB groups or to cluster proteomes into new ortholog groups. Curr Protoc Bioinformatics. 2011:6–12. doi: 10.1002/0471250953.bi0612s35. PMID: 21901743 10.1002/0471250953.bi0612s35 PMC3196566 21901743

[32] Sirko A, Zatyka M, Sadowy E, Hulanicka D (1995). Sulfate and thiosulfate transport in *Escherichia coli* K-12: evidence for a functional overlapping of sulfate- and thiosulfate-binding proteins. J Bacteriol.

[33] Sekowska A, Kung HF, Danchin A (2000). Sulfur metabolism in *Escherichia coli* and related bacteria: facts and fiction. J Mol Microbiol Biotechnol.

[34] Ackerley DF, Gonzalez CF, Park CH, Blake R, Keyhan M, Matin A (2004). Chromate-reducing properties of soluble flavoproteins from *Pseudomonas putida* and *Escherichia coli*. Appl Environ Microbiol.

[35] Branco R, Chung AP, Johnston T, Gurel V, Morais P, Zhitkovich A (2008). The chromate-inducible *chrBACF* operon from the transposable element *TnOtChr* confers resistance to chromium (VI) and superoxide. J Bacteriol.

[36] Henne KL, Nakatsu CH, Thompson DK, Konopka AE (2009). High-level chromate resistance in *Arthrobacter* sp. strain FB24 requires previously uncharacterized accessory genes. BMC Microbiol.

[37] Makui H, Roig E, Cole ST, Helmann JD, Gros P, Cellier MF (2000). Identification of the *Escherichia coli* K-12 Nramp orthologue (MntH) as a selective divalent metal ion transporter. Mol Microbiol.

[38] Nies DH, Nies A, Chu L, Silver S (1989). Expression and nucleotide sequence of a plasmid-determined divalent cation efflux system from *Alcaligenes eutrophus*. Proc Natl Acad Sci U S A.

[39] Rensing C, Mitra B, Rosen BP (1997). The *zntA* gene of *Escherichia coli* encodes a Zn (II)-translocating P-type ATPase. Proc Natl Acad Sci U S A.

[40] Odermatt A, Suter H, Krapf R, Solioz M (1993). Primary structure of two P-type ATPases involved in copper homeostasis in *Enterococcus hirae*. J Biol Chem.

[41] Adaikkalam V, Swarup S (2005). Characterization of *copABCD* operon from a copper-sensitive *Pseudomonas putida* strain. Can J Microbiol.

[42] Kruger MC, Bertin PN, Heipieper HJ, Arsène-Ploetze F (2013). Bacterial metabolism of environmental arsenic--mechanisms and biotechnological applications. Appl Microbiol Biotechnol.

[43] Rosen BP, Ajees AA, McDermott TR (2011). Life and death with arsenic. Arsenic life: an analysis of the recent report “A bacterium that can grow by using arsenic instead of phosphorus”. Bioessays.

[44] Nascimento AM, Chartone-Souza E (2003). Operon mer: bacterial resistance to mercury and potential for bioremediation of contaminated environments. Genet Mol Res.

[45] Tamura K, Stecher G, Peterson D, Filipski A, Kumar S (2013). MEGA6: molecular evolutionary genetics analysis version 6.0. Mol Biol Evol.

[46] Monteiro MI, Fraga IC, Yallouz AV, de Oliveira NM, Ribeiro SH (2002). Determination of total chromium traces in tannery effluents by electrothermal atomic absorption spectrometry, flame atomic absorption spectrometry and UV-visible spectrophotometric methods. Talanta.

[47] Field D, Garrity GM, Gray T, Morrison N, Selengut J, Sterk P (2008). The minimum information about a genome sequence (MIGS) specification. Nat Biotechnol.

[48] Woese CR, Kandler O, Weelis ML (1990). Towards a natural system of organisms: proposal for the domains archaea, bacteria and eucarya. Proc Natl Acad Sci U S A.

[49] Garrity GM, Bell JA, Phylum Lilburn T, Brenner DJ, Krieg NR, Stanley JT, Garrity GM, XIV (2005). *Proteobacteria* phyl nov. Bergey’s Manual of Sytematic Bacteriology, second edition. Vol. 2 (The *Proteobacteria*), part B (The *Gammaproteobacteria*).

[50] Stackebrandt E, Murray RGE, Trüper HG (1988). *Proteobacteria* classis nov., a name for the phylogenetic taxon that includes the “purple bacteria and their relatives”. Int J Syst Evol Microbiol.

[51] Garrity GM, Bell JA, Class LT, Brenner DJ, Krieg NR, Stanley JT, Garrity GM, III (2005). Gammaproteobacteria class. nov. Bergey’s Manual of Sytematic Bacteriology, second edition. Vol. 2 (The *Proteobacteria*), part B (The *Gammaproteobacteria*).

[52] Validation of publication of new names and newcombinations previously effectively published outside the IJSEM. List no. 106. Int J Syst Evol Microbiol. 2005;55:2235–38. 10.1099/ijs.0.63767-0 15879221

[53] Williams KP, Kelly DP (2013). Proposal for a new class within the phylum *Proteobacteria*, *Acidithiobacillia* classis nov., with the type order *Acidithiobacillales*, and emended description of the class *Gammaproteobacteria*. Int J Syst Evol Microbiol.

[54] Bowman JP, Mcmeekin TA, Order X, Brenner DJ, Krieg NR, Stanley JT, Garrity GM (2005). Alteromonadales ord. nov. Bergey’s Manual of Sytematic Bacteriology, second edition. Vol. 2 (The *Proteobacteria*), part B (The *Gammaproteobacteria*).

[55] Ivanova EP, Mikhaĭlov VV (2001). A new family of *Alteromonadaceae* fam. nov., including the marine *proteobacteria* species *Alteromonas*, *Pseudoalteromonas*, *Idiomarina* and *Colwellia*. Microbiology.

[56] Ashburner M, Ball CA, Blake JA, Botstein D, Butler H, Cherry JM (2000). Gene ontology: tool for the unification of biology. The gene ontology consortium. Nat Genet.

