# Supplementary material for: Draft genomic sequence of a chromate- and sulfate-reducing Alishewanella strain with the ability to bioremediate Cr and Cd contamination
Source: Stand Genomic Sci. 2016 Aug 5;11:48. doi: 10.1186/s40793-016-0169-3 (PMC4974768; doi:10.1186/s40793-016-0169-3)
Supplement: Additional file 1: — Figure S1. Phylogenetic relationships of Alishewanella sp. WH16-1 based on gyrB sequences. The analysis was performed by MEGA 6.0 [45] with NJ algorithm and 1,000 bootstrap repetitions were computed to estimate the reliability of the tree. The gyrB gene of strain WH16-1 is the gene sequence coding for AAY72_13600. Figure S2. The putative sulfate transport and reduction pathway in Alishewanella sp. WH16-1. APS stands for adenylylsulphate, PAPS stands for phosphoadenylylsulphate. The locus tag numbers of the predicted proteins (CysP, CysU, CysW, CysA, CysN, CysD, CysC, CysH CysJ and CysI) are AAY72_14890, AAY72_14885, AAY72_14880, AAY72_14875, AAY72_03865, AAY72_03870, AAY72_07290, AAY72_07265, AAY72_07255 and AAY72_07260, respectively. (DOCX 355 kb) [file 40793_2016_169_MOESM1_ESM.docx]

**Draft genomic sequence of a chromate- and** **sulfate-reducing *Alishewanella* with the ability to bioremediate Cr and Cd contamination**

Xian Xia^1^, Jiahong Li^1^, Shuijiao Liao^1, 2^, Gaoting Zhou^1, 2^, Hui Wang^1^, Liqiong Li^1^, Biao Xu^1^, Gejiao Wang^1^*

^1^ State Key Laboratory of Agricultural Microbiology, ^2^ College of Basic Sciences, Huazhong Agricultural University, Wuhan, 430070, P. R. China

*Correspondence should be addressed to: Dr. Gejiao Wang, Tel: +86 27 87281261, Fax: +86 27 87280670, E-mail: [gejiao@mail.hzau.edu.cn](mailto:gejiao@mail.hzau.edu.cn)


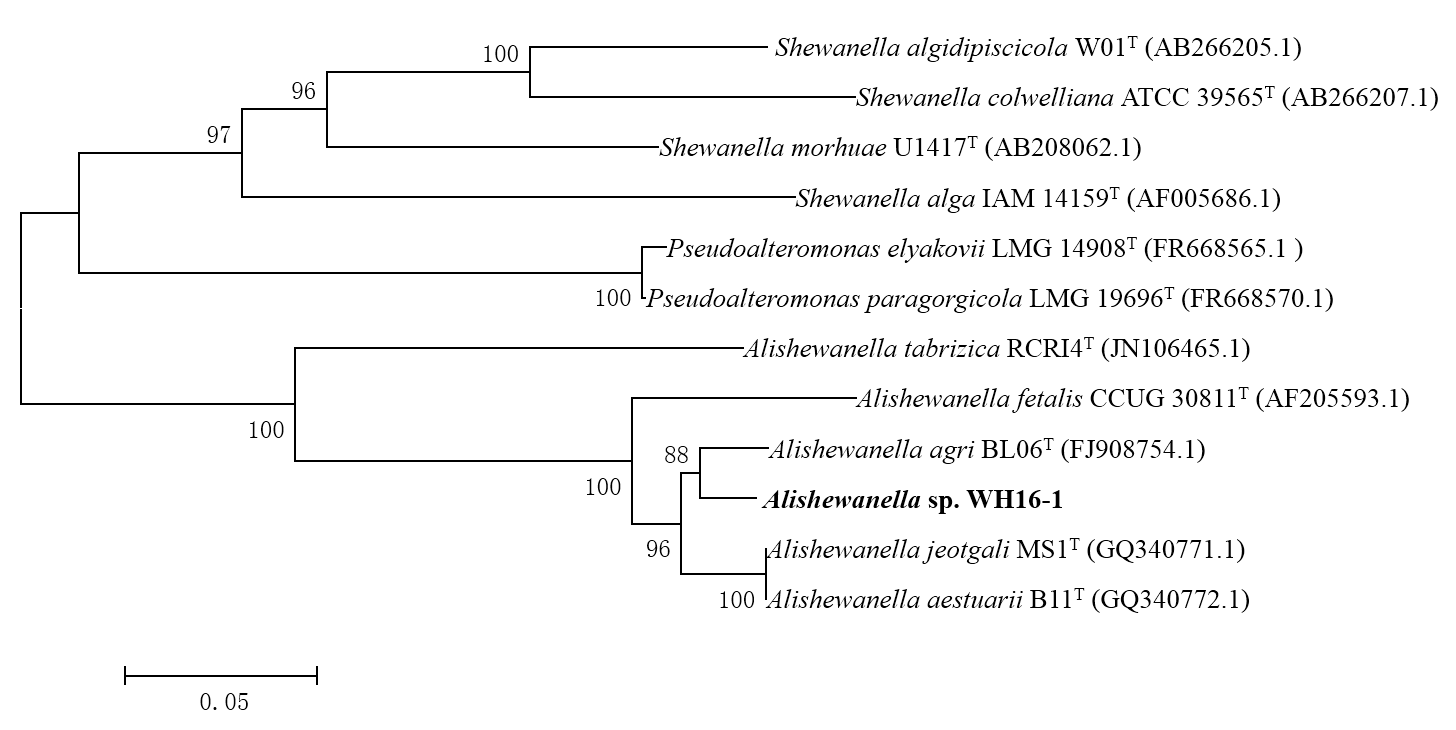


**Fig. S1** Phylogenetic relationships of *Alishewanella* sp. WH16-1 based on *gyrB* sequences. The analysis was performed by MEGA 6.0 [13] with NJ algorithm and 1,000 bootstrap repetitions were computed to estimate the reliability of the tree. The *gyrB* gene of strain WH16-1 is the gene sequence coding for AAY72_13600.


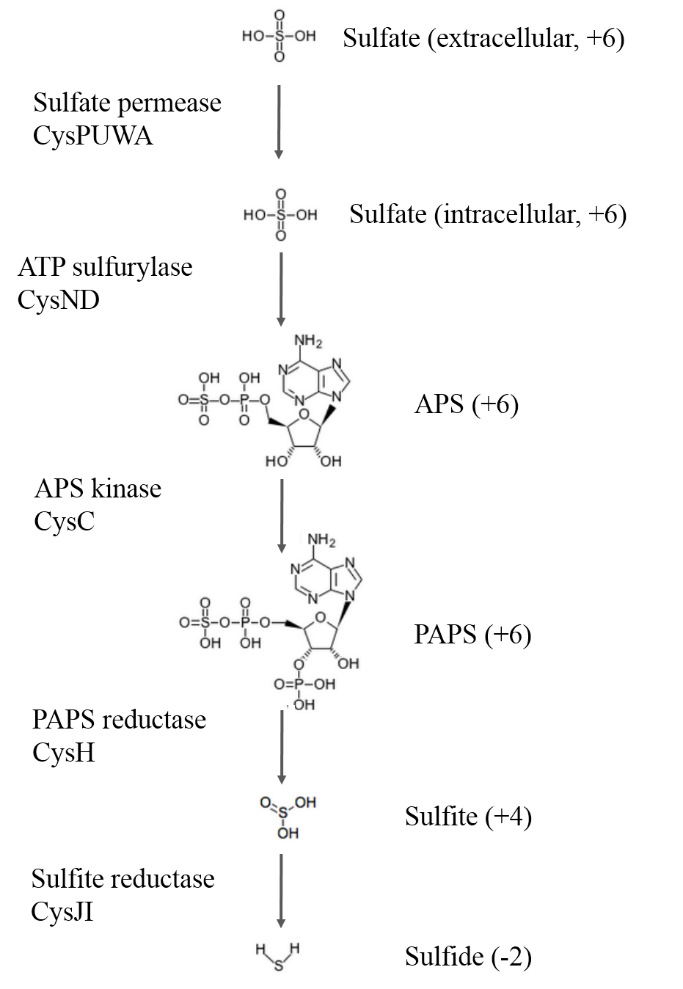


**Fig. S2** The putative sulfate transport and reduction pathway in *Alishewanella* sp. WH16-1. APS stands for adenylylsulphate, PAPS stands for phosphoadenylylsulphate. The locus tag numbers of the predicted proteins (CysP, CysU, CysW, CysA, CysN, CysD, CysC, CysH CysJ and CysI) are AAY72_14890, AAY72_14885, AAY72_14880, AAY72_14875, AAY72_03865, AAY72_03870, AAY72_07290, AAY72_07265, AAY72_07255 and AAY72_07260, respectively.
